# Predictive value of stress hyperglycemia ratio for the risk of heart failure during hospitalization in STEMI patients undergoing emergency percutaneous coronary intervention: a single-center retrospective cohort study

**DOI:** 10.3389/fcvm.2026.1819191

**Published:** 2026-08-18

**Authors:** Fachao Shi, Wenlong Ding, Cunming Fang, Caoyang Fang

**Affiliations:** 1 Department of Cardiology, Maanshan People's Hospital of Wannan Medical University, Maanshan, Anhui, China; 2 Department of Cardiology, Xuancheng Hospital Affiliated to Wannan Medical College (Xuancheng People’s Hospital), Xuancheng, Anhui, China; 3 Department of Emergency, the First Affiliated Hospital of USTC, Division of Life Sciences and Medicine, University of Science and Technology of China, Hefei, Anhui, China

**Keywords:** heart failure, percutaneous coronary intervention, SHR, ST-elevation myocardial infarction, STEMI, stress hyperglycemia ratio

## Abstract

**Background:**

Stress hyperglycemia is prevalent in acute ST-elevation myocardial infarction (STEMI) patients and associates with adverse outcomes. The stress hyperglycemia ratio (SHR), which standardizes admission glucose against estimated average glucose from HbA1c, reduces chronic glycemic interference. However, SHR's predictive value for heart failure (HF) during hospitalization following emergency percutaneous coronary intervention (PCI) remains unclear.

**Methods:**

This single-center retrospective cohort study enrolled 1,068 consecutive STEMI patients undergoing emergency PCI from January 2020 to June 2025. SHR was calculated as the ratio of admission plasma glucose to estimated average glucose. The primary endpoint was hospitalization HF. Predictive efficacy was evaluated using multivariate logistic regression, propensity score matching (PSM), receiver operating characteristic (ROC) analysis, net reclassification improvement (NRI), and integrated discrimination improvement (IDI).

**Results:**

Overall, 158 patients (14.8%) developed hospitalization HF. The HF group showed significantly higher SHR than non-HF patients [1.52 (1.25–1.88) vs. 1.10 (0.92–1.30); *P* < 0.001]. Each 1-unit SHR increase associated with 2.31-fold HF risk elevation (adjusted OR=2.31, 95% CI 1.68–3.18; *P* < 0.001). Following 1:1 PSM, high SHR patients maintained significantly higher HF incidence (20.4% vs. 10.2%; *P* < 0.001). SHR's ROC area under curve was 0.748, superior to admission glucose (0.728; *P* = 0.031). Adding SHR yielded NRI = 0.318 and IDI = 0.035 (both *P* < 0.001).

**Conclusions:**

SHR independently predicts hospitalization HF in STEMI patients post-emergency PCI, demonstrating superior discriminative performance to admission glucose with significant incremental predictive value. SHR may facilitate early risk stratification and management.

## 1 Introduction

ST-elevation myocardial infarction (STEMI) represents the most critical type of acute coronary syndrome. Although emergency percutaneous coronary intervention (PCI) and contemporary pharmacological therapies have substantially reduced in-hospital mortality, heart failure (HF) during hospitalization remains one of the most frequent severe complications in STEMI patients, with incidence rates ranging from 14% to 36% (1–3). The occurrence of HF not only prolongs hospital stay and increases healthcare costs but also serves as an independent and powerful predictor of both short-term and long-term mortality (4, 5). Therefore, early identification of high-risk populations for HF development upon admission is of paramount importance for optimizing monitoring levels, guiding hemodynamic management, and improving prognosis.

Stress hyperglycemia refers to transient glucose elevation caused by activation of the sympathetic-adrenal axis and hypothalamic-pituitary-adrenal (HPA) axis during acute illness, leading to massive release of counter-regulatory hormones (cortisol, catecholamines, glucagon, and growth hormone). This phenomenon occurs in patients both with and without a history of diabetes (6, 7). Extensive previous research has confirmed that elevated admission glucose is independently associated with increased infarct size, reduced myocardial salvage, and higher in-hospital mortality and major adverse cardiovascular event (MACE) risk in acute myocardial infarction (AMI) patients (8–10).

However, absolute admission glucose levels as indicators of stress severity have inherent limitations: admission glucose simultaneously reflects both acute stress response and patients’ baseline chronic glycemic status. For diabetic patients with chronically poor glucose control, “elevated” admission glucose may represent only a mild stress response, whereas the same absolute value in non-diabetic patients indicates profound stress (11). This “baseline glucose confounding effect” severely undermines the clinical interpretability of admission glucose as an acute stress biomarker.

To overcome these limitations, Roberts et al. introduced the concept of stress hyperglycemia ratio (SHR) in 2015 (12). SHR is calculated as admission glucose divided by estimated average glucose (eAG), where eAG (mmol/L) = 1.59 × HbA1c (%)−2.59 (13). By standardizing admission glucose to individual chronic glycemic levels, SHR eliminates interference from baseline glucose metabolism and more precisely reflects the relative magnitude of acute stress-induced glucose elevation. In recent years, the prognostic value of SHR has been preliminarily validated in critical care scenarios including acute coronary syndrome, acute ischemic stroke, sepsis, and cardiac surgery (14–17).

Based on this background, the present retrospective cohort study aims to investigate the independent association between SHR and HF risk during hospitalization following emergency PCI in STEMI patients.

## 2 Methods

### 2.1 Study design and population

This study was a single-center, retrospective cohort study that consecutively enrolled patients who underwent emergency PCI for STEMI in the Department of Cardiology at Anhui Provincial Hospital from January 2020 to June 2025.Inclusion criteria:① Age ≥18 years; ② Meeting the diagnostic criteria for STEMI according to the Fourth Universal Definition of Myocardial Infarction (18); ③Receiving emergency PCI with a standard indication for STEMI reperfusion therapy, with symptom onset to balloon inflation time strictly limited within 12 h. Exclusion criteria:① Previous chronic heart failure (NYHA Class II–IV); Notably, only patients with symptomatic chronic heart failure were excluded, and patients with asymptomatic left ventricular dysfunction were included in the present study. Left ventricular ejection fraction (LVEF) was fully adjusted in the multivariate regression model to control for the confounding effect of baseline asymptomatic left ventricular systolic dysfunction; ② Severe valvular heart disease; ③ Cardiogenic shock requiring mechanical circulatory support prior to PCI upon admission; ④ Missing admission glucose or HbA1c data; ⑤ HbA1c < 4.0% or >14.0% (extreme values suggesting measurement error); ⑥ Active malignancy or life expectancy <6 months; ⑦ Severe hepatic insufficiency (Child-Pugh Class C).The study protocol was approved by the Ethics Committee of Anhui Provincial Hospital and was conducted in accordance with the Declaration of Helsinki. Given the retrospective nature of the study, individual informed consent was waived.

### 2.2 Data collection

All data were extracted from the hospital electronic medical records (HIS) system. The recorded variables included the following categories:Demographics and medical history: Age, sex, body mass index (BMI), smoking history, alcohol consumption history, hypertension, diabetes mellitus, dyslipidemia, previous ischemic stroke, previous myocardial infarction, and previous PCI history. Admission vital signs: Heart rate, systolic blood pressure (SBP), and diastolic blood pressure (DBP). Laboratory parameters on admission and during hospitalization: Admission plasma glucose (emergency blood sampling), glycated hemoglobin (HbA1c), peak cardiac troponin I (cTnI), N-terminal pro-B-type natriuretic peptide (NT-proBNP), serum creatinine (Scr), estimated glomerular filtration rate (eGFR, CKD-EPI equation), white blood cell (WBC) count, hemoglobin (Hb),platelet count (PLT),total cholesterol (TC), low-density lipoprotein cholesterol (LDL-C), high-density lipoprotein cholesterol (HDL-C), triglycerides (TG), serum potassium, serum sodium, uric acid (UA),D-dimer, and high-sensitivity C-reactive protein (hs-CRP). Echocardiographic parameters: Left ventricular ejection fraction (LVEF) measured by the first transthoracic echocardiography performed after hospital admission and prior to the occurrence of in-hospital heart failure events during hospitalization. Coronary angiography and procedural variables: Culprit vessel (LAD, LCX, RCA, or LM), number of diseased vessels (single-vessel vs. multivessel disease), door-to-balloon time (D-to-B), and post-procedural TIMI flow grade. All data were extracted from the hospital electronic medical records (HIS) system. The recorded variables included the following categories: Demographics and medical history: Age, sex, body mass index (BMI), smoking history, alcohol consumption history, hypertension, diabetes mellitus, dyslipidemia, previous ischemic stroke, previous myocardial infarction, and previous PCI history. Admission vital signs: Heart rate, systolic blood pressure (SBP), and diastolic blood pressure (DBP). Laboratory parameters on admission and during hospitalization: Admission plasma glucose (emergency blood sampling), glycated hemoglobin (HbA1c), peak cardiac troponin I (cTnI), N-terminal pro-B-type natriuretic peptide (NT-proBNP), serum creatinine (Scr), estimated glomerular filtration rate (eGFR, CKD-EPI equation), white blood cell (WBC) count, hemoglobin (Hb),platelet count (PLT),total cholesterol (TC),low-density lipoprotein cholesterol (LDL-C),high-density lipoprotein cholesterol (HDL-C), triglycerides (TG),serum potassium, serum sodium, uric acid (UA),D-dimer, and high-sensitivity C-reactive protein (hs-CRP). Echocardiographic parameters: Left ventricular ejection fraction (LVEF) measured by the first transthoracic echocardiography performed after hospital admission and prior to the occurrence of in-hospital heart failure events during hospitalization. Coronary angiography and procedural variables: Culprit vessel (LAD, LCX, RCA, or LM), number of diseased vessels (single-vessel vs. multivessel disease), door-to-balloon time (D-to-B), and post-procedural TIMI flow grade. In-hospital medications: Aspirin, P2Y₁₂ receptor inhibitors, statins, *β*-blockers, ACE inhibitors/angiotensin receptor blockers (ACEI/ARB), and diuretics. Mechanical support devices: Intra-aortic balloon pump (IABP). Mechanical support devices: Intra-aortic balloon pump (IABP).

### 2.3 Calculation of stress-induced hyperglycemia ratio

SHR was calculated using the formula established by Roberts et al. (12): SHR = admission glucose (mmol/L)/eAG (mmol/L). Estimated average glucose (eAG) was derived from the HbA1c-to-average glucose conversion formula published by Nathan et al. (13): eAG (mmol/L) = 1.59 × HbA1c (%)−2.59. An SHR of 1.0 indicates that admission glucose equals the chronic average glucose level; SHR > 1.0 suggests acute stress-induced hyperglycemia above baseline glucose levels; SHR < 1.0 indicates relative hypoglycemia or acute-phase glucose below baseline levels.

### 2.4 Definition of primary endpoints

The primary endpoint was definite new-onset or deteriorated in-hospital acute heart failure (AHF) after emergency PCI, which was strictly defined based on standardized clinical manifestations, objective imaging evidence, and standardized therapeutic intervention to reduce phenotypic heterogeneity of study endpoints. Notably, the study endpoint focused on post-PCI incident HF events, excluding persistent heart failure status that existed stably before emergency PCI. The diagnostic criteria must meet at least one of the following objective and confirmatory conditions during hospitalization after PCI: ① New-onset heart failure corresponding to Killip class ≥ II after STEMI, or deterioration of heart failure with Killip grade upgrade compared with pre-PCI baseline status (19); ② Typical acute heart failure signs and symptoms (orthopnea, paroxysmal nocturnal dyspnea, bilateral pulmonary rales involving more than half of the lung fields, obvious jugular venous distension, or peripheral edema) newly occurring after PCI, accompanied by initiation or intravenous intensification of diuretic/vasodilator therapy for HF treatment; ③ Newly emerged objective chest imaging evidence of pulmonary congestion or pulmonary edema consistent with acute heart failure after PCI; ④ New-onset cardiogenic shock secondary to post-PCI myocardial dysfunction (persistent systolic blood pressure <90 mmHg for more than 30 min with end-organ hypoperfusion, requiring inotropic or vasopressor support), which represents severe decompensated heart failure (20, 21).

To ensure endpoint consistency and reduce adjudication bias: Patients with only mild, non-specific dyspnea without objective imaging evidence or standardized HF treatment intervention were excluded from endpoint events. Patients with stable baseline Killip class II before PCI who maintained persistent and unimproved but non-aggravated Killip class II status after PCI without any HF deterioration or new intervention were not defined as endpoint events. All in-hospital HF events were independently adjudicated by two senior attending cardiologists. Any discrepancies in judgment were resolved by discussion and final confirmation by a deputy chief physician specializing in cardiovascular diseases to guarantee the accuracy and homogeneity of endpoint inclusion. Notably, baseline Killip class II–IV heart failure at admission (pre-PCI) that was significantly relieved and improved after PCI and standardized medical treatment, without post-PCI HF deterioration or new HF events, was not adjudicated as an endpoint event, which ensures consistent matching between baseline clinical characteristics and outcome definition.

### 2.5 Statistical analysis

Normality of continuous variables was assessed using the Shapiro–Wilk test combined with histograms and Q-Q plots. Normally distributed continuous variables were expressed as mean ± standard deviation (x¯ ± SD) and compared using independent-sample t-tests; non-normally distributed variables were expressed as median (interquartile range, IQR) and compared using the Mann–Whitney U test. Categorical variables were expressed as frequencies (percentages) and compared using *χ*^2^ tests or Fisher's exact tests. Univariate logistic regression was performed first, with variables showing *P* < 0.05 entered into the multivariate model. Two models were constructed: Model 1 included SHR as a continuous variable; Model 2 categorized SHR into tertiles [T1 (reference), T2, T3] and tested for trend (*P* for trend). Multicollinearity was assessed using variance inflation factors (VIF > 5 indicating severe collinearity). Model calibration was comprehensively evaluated by both statistical testing and visual verification. The Hosmer-Lemeshow goodness-of-fit test was performed for quantitative calibration assessment. Additionally, a calibration curve was plotted by stratifying patients into 10 equal risk deciles according to model-predicted in-hospital HF probability, to visually compare the consistency between mean predicted probabilities and actual observed event rates across different risk strata.

Patients were dichotomized into high and low SHR groups based on the median SHR value. Propensity scores were calculated using logistic regression with baseline covariates as independent variables. One-to-one nearest-neighbor matching without replacement was performed using a caliper width of 0.2 times the standard deviation of the logit of the propensity score. Post-matching covariate balance was considered adequate when absolute standardized mean differences (SMD) were <0.10 (22). HF incidence was compared between groups after matching. Using the rcs function from the rms package, multivariate-adjusted logistic regression with restricted cubic splines was fitted using four knots positioned at the 5th, 35th, 65th, and 95th percentiles of SHR distribution. The median SHR was used as the reference value to explore dose-response relationships between SHR and HF risk. Statistical significance was assessed using likelihood ratio tests.

ROC curves were plotted for SHR, admission glucose, HbA1c, and the multivariate logistic model predicting HF, with area under the curve (AUC, C-statistic) calculated. AUC differences between predictors were compared using the DeLong method. The optimal SHR cutoff value was determined using the Youden index, with corresponding sensitivity, specificity, positive predictive value (PPV), and negative predictive value (NPV) reported. A basic clinical model (containing conventional risk factors but excluding SHR) was constructed. After adding SHR to the basic model, incremental predictive value was quantified using likelihood ratio tests (LRT), *Δ*AUC (DeLong method), category-free net reclassification improvement (NRI), and integrated discrimination improvement (IDI) (23). Akaike information criterion (AIC) and Bayesian information criterion (BIC) were compared between models containing SHR, admission glucose, and the basic model.

Stratified analyses were performed by age (<65 vs. ≥ 65 years), sex, diabetes status, Killip class (I–II vs. III–IV), LVEF (<50% vs. ≥ 50%), number of diseased vessels (single-vessel vs. multivessel disease), and renal function (eGFR <60 vs. ≥ 60 mL/min/1.73 m^2^). Interactions between SHR and stratification variables were tested using multiplicative interaction terms. Sensitivity analyses included: ① Separate analyses in diabetic and non-diabetic subgroups; ② Repeating the primary analysis after excluding Killip class IV patients; ③ Repeating the primary analysis after excluding patients with eGFR <30 mL/min/1.73 m^2^; ④ Comparing AIC and BIC between models containing SHR versus admission glucose. All statistical analyses were performed using R software (version 4.3.1). Two-sided *P* < 0.05 was considered statistically significant.

## 3 Results

### 3.1 Baseline characteristics

A total of 1,068 patients were included in this study. All baseline clinical characteristics analyzed in this section were collected at hospital admission and before emergency PCI. In-hospital medication data were collected during post-PCI hospitalization. Among them, 158 patients (14.8%) experienced new-onset or deteriorated heart failure during hospitalization after emergency PCI, while 910 patients (85.2%) did not develop post-PCI HF events. Stratified breakdown of the composite in-hospital HF endpoints among 158 event patients was further performed: 117 patients presented with mild-to-moderate HF events corresponding to new-onset or deteriorated Killip class II–III heart failure with clinical pulmonary congestion and required intensified diuretic or vasodilator therapy; 41 patients developed severe HF events, including Killip class IV heart failure and cardiogenic shock requiring vasopressor/inotropic support or mechanical circulatory assistance. Severe HF events accounted for 25.9% of all in-hospital HF cases, indicating that elevated SHR was predictive of both mild and severe post-PCI HF complications across a broad clinical severity spectrum. Baseline characteristics and in-hospital treatment information stratified by in-hospital HF status are shown in Table 1. Compared with the non-HF group, patients in the HF group were older (67.3 ± 11.5 vs. 60.8 ± 12.2 years; *P* < 0.001), had a higher proportion of females (31.6% vs. 21.8%; *P* = 0.008), and higher prevalence of hypertension (62.0% vs. 48.1%; *P* = 0.001) and diabetes mellitus (38.0% vs. 24.2%; *P* < 0.001). At admission, the HF group had faster heart rate (88.5 ± 18.2 vs. 76.3 ± 14.5 bpm), lower systolic blood pressure (118.5 ± 24.3 vs. 128.6 ± 21.5 mmHg), and lower LVEF (42.8 ± 9.5% vs. 55.6 ± 8.2%). A higher proportion had LAD as the culprit vessel (52.5% vs. 37.8%), multivessel disease was more common (58.2% vs. 41.5%), and D-to-B time was longer [median 120 (82–178) vs. 95 (66–138) min].Regarding the core exposure variables, the HF group had significantly higher admission glucose [median 10.8 (8.2–14.5) vs. 7.5 (5.9–9.6) mmol/L; *P* < 0.001], higher HbA1c (6.5 ± 1.6% vs. 6.1 ± 1.2%; *P* = 0.002), and markedly elevated SHR [median 1.52 (1.25–1.88) vs. 1.10 (0.92–1.30); *P* < 0.001]. The HF group also showed significantly higher levels of peak cTnI, NT-proBNP, WBC, hs-CRP, D-dimer, and UA, while eGFR, Hb, HDL-C, and serum sodium were lower (all *P* < 0.05).

**Table 1:** Baseline characteristics stratified by heart failure status during hospitalization.

Variables	Total (*n* = 1,068)	HF group (*n* = 158)	Non-HF group (*n* = 910)	*P*-value
Age (years)	61.8 ± 12.2	67.3 ± 11.5	60.8 ± 12.2	< 0.001
Male, *n*(%)	820 (76.8)	108 (68.4)	712 (78.2)	0.008
BMI (kg/m^2^)	24.7 ± 3.2	25.1 ± 3.4	24.6 ± 3.2	0.085
Smoke, *n*(%)	548 (51.3)	76 (48.1)	472 (51.9)	0.383
Alcohol, *n*(%)	258 (24.2)	35 (22.2)	223 (24.5)	0.517
Hypertension, *n*(%)	536 (50.2)	98 (62.0)	438 (48.1)	0.001
Diabetes, *n*(%)	280 (26.2)	60 (38.0)	220 (24.2)	< 0.001
Dyslipidemia, *n*(%)	328 (30.7)	55 (34.8)	273 (30.0)	0.223
Previous stroke, *n*(%)	81 (7.6)	19 (12.0)	62 (6.8)	0.023
Previous PCI, *n*(%)	90 (8.4)	16 (10.1)	74 (8.1)	0.409
Previous MI, *n*(%)	60 (5.6)	14 (8.9)	46 (5.1)	0.051
Heart rate (bpm)	78.1 ± 15.5	88.5 ± 18.2	76.3 ± 14.5	< 0.001
SBP (mmHg)	127.1 ± 22.2	118.5 ± 24.3	128.6 ± 21.5	< 0.001
DBP (mmHg)	77.6 ± 12.5	72.3 ± 13.8	78.5 ± 12.2	< 0.001
Killip grade, *n*(%)				< 0.001
Ⅰ	702 (65.7)	47 (29.7)	655 (72.0)	
Ⅱ	222 (20.8)	56 (35.4)	166 (18.2)	
Ⅲ	99 (9.3)	35 (22.2)	64 (7.0)	
Ⅳ	45 (4.2)	20 (12.7)	25 (2.7)	
LVEF (%)	53.7 ± 9.6	42.8 ± 9.5	55.6 ± 8.2	< 0.001
Admission blood glucose (mmol/L)^a^	7.8 (6.1–10.5)	10.8 (8.2–14.5)	7.5 (5.9–9.6)	< 0.001
HbA1c (%)	6.2 ± 1.3	6.5 ± 1.6	6.1 ± 1.2	0.002
SHR^a^	1.14 (0.94–1.40)	1.52 (1.25–1.88)	1.10 (0.92–1.30)	< 0.001
Peak cTnI (ng/mL)^a^	15.2 (7.8–30.5)	35.6 (18.2–58.3)	12.8 (6.5–25.6)	< 0.001
NT-proBNP (pg/mL)^a^	568 (185–2 150)	3 520 (1 680–6 850)	448 (152–1 280)	< 0.001
Scr (*μ*mol/L)^a^	80.2 (66.5–98.3)	95.5 (72.8–125.6)	78.2 (65.2–94.5)	< 0.001
eGFR (mL/min/1.73 m^2^)	85.6 ± 23.8	72.5 ± 26.2	87.9 ± 22.5	< 0.001
WBC (×10⁹/L)^a^	10.0 (7.8–12.5)	11.5 (9.2–14.8)	9.8 (7.6–12.2)	< 0.001
Hb (g/L)	136.8 ± 17.0	128.5 ± 18.3	138.2 ± 16.5	< 0.001
PLT (×10⁹/L)	215 ± 62	212 ± 65	216 ± 61	0.452
TC (mmol/L)	4.65 ± 1.10	4.72 ± 1.15	4.64 ± 1.09	0.408
LDL-C (mmol/L)	2.81 ± 0.89	2.95 ± 0.92	2.78 ± 0.88	0.028
HDL-C (mmol/L)	1.10 ± 0.30	1.02 ± 0.28	1.12 ± 0.30	< 0.001
TG (mmol/L)^a^	1.55 (1.02–2.28)	1.58 (1.05–2.35)	1.54 (1.02–2.25)	0.562
Blood potassium (mmol/L)	4.05 ± 0.50	4.08 ± 0.52	4.04 ± 0.49	0.348
Blood sodium (mmol/L)	140.0 ± 3.3	138.5 ± 3.8	140.2 ± 3.2	< 0.001
UA (μmol/L)	347 ± 97	385 ± 105	340 ± 95	< 0.001
hs-CRP (mg/L)^a^	8.5 (3.8–18.5)	18.2 (9.5–35.6)	7.8 (3.5–16.2)	< 0.001
D-dimer (mg/L)^a^	0.85 (0.42–1.68)	1.82 (0.95–3.28)	0.78 (0.38–1.52)	< 0.001
Culprit vessel, *n*(%)				0.001
LAD	427 (40.0)	83 (52.5)	344 (37.8)	
LCX	152 (14.2)	19 (12.0)	133 (14.6)	
RCA	436 (40.8)	47 (29.7)	389 (42.7)	
LM	53 (5.0)	9 (5.7)	44 (4.8)	
Multivessel disease, *n*(%)	470 (44.0)	92 (58.2)	378 (41.5)	< 0.001
D-to-B time (min)^a^	98 (68–145)	120 (82–178)	95 (66–138)	< 0.001
TIMI 3 POST PCI, *n*(%)	926 (86.7)	119 (75.3)	807 (88.7)	< 0.001
Aspirin, *n*(%)	1 035 (96.9)	150 (94.9)	885 (97.3)	0.115
P2Y₁₂ inhibitor, *n*(%)	1 025 (96.0)	148 (93.7)	877 (96.4)	0.105
Statins, *n*(%)	1 013 (94.9)	145 (91.8)	868 (95.4)	0.068
Beta blockers, *n*(%)	859 (80.4)	114 (72.2)	745 (81.9)	0.005
ACEI/ARB, *n*(%)	672 (62.9)	107 (67.7)	565 (62.1)	0.168
Diuretics, *n*(%)	278(26.0)	114(72.2)	164(18.0)	< 0.001
IABP, *n*(%)	51(4.8)	24(15.2)	27(3.0)	< 0.001

a Non-normally distributed variables are expressed as median (IQR).

BMI, Body Mass Index; SBP, Systolic Blood Pressure; DBP, Diastolic Blood Pressure; LVEF, Left Ventricular Ejection Fraction; LAD, Left Anterior Descending artery; LCX, Left Circumflex artery; RCA, Right Coronary Artery; LM, Left Main Coronary Artery; D-to-B, Door-to-Balloon Time; TIMI, Thrombolysis In Myocardial Infarction; IABP, Intra-Aortic Balloon Pump.

All baseline data (demographics, medical history, admission vital signs, laboratory indexes, and angiographic characteristics) were collected at hospital admission and before emergency PCI. All in-hospital medication data and IABP application status were recorded during hospitalization after emergency PCI. In the Non-HF group, patients with baseline Killip class II–IV had acute heart failure symptoms caused by STEMI at admission; all these patients achieved significant symptomatic improvement and Killip class regression after PCI and in-hospital treatment, without post-procedural new-onset heart failure or aggravated HF events. The 18% diuretic use rate in the Non-HF group was attributed to low-dose prophylactic diuretic therapy for mild post-STEMI volume overload or mild pulmonary congestion; these patients did not receive intensified HF treatment and did not meet the diagnostic criteria for study-defined in-hospital acute heart failure endpoints.

### 3.2 SHR tertiles and incidence of heart failure

**Figure 1:**
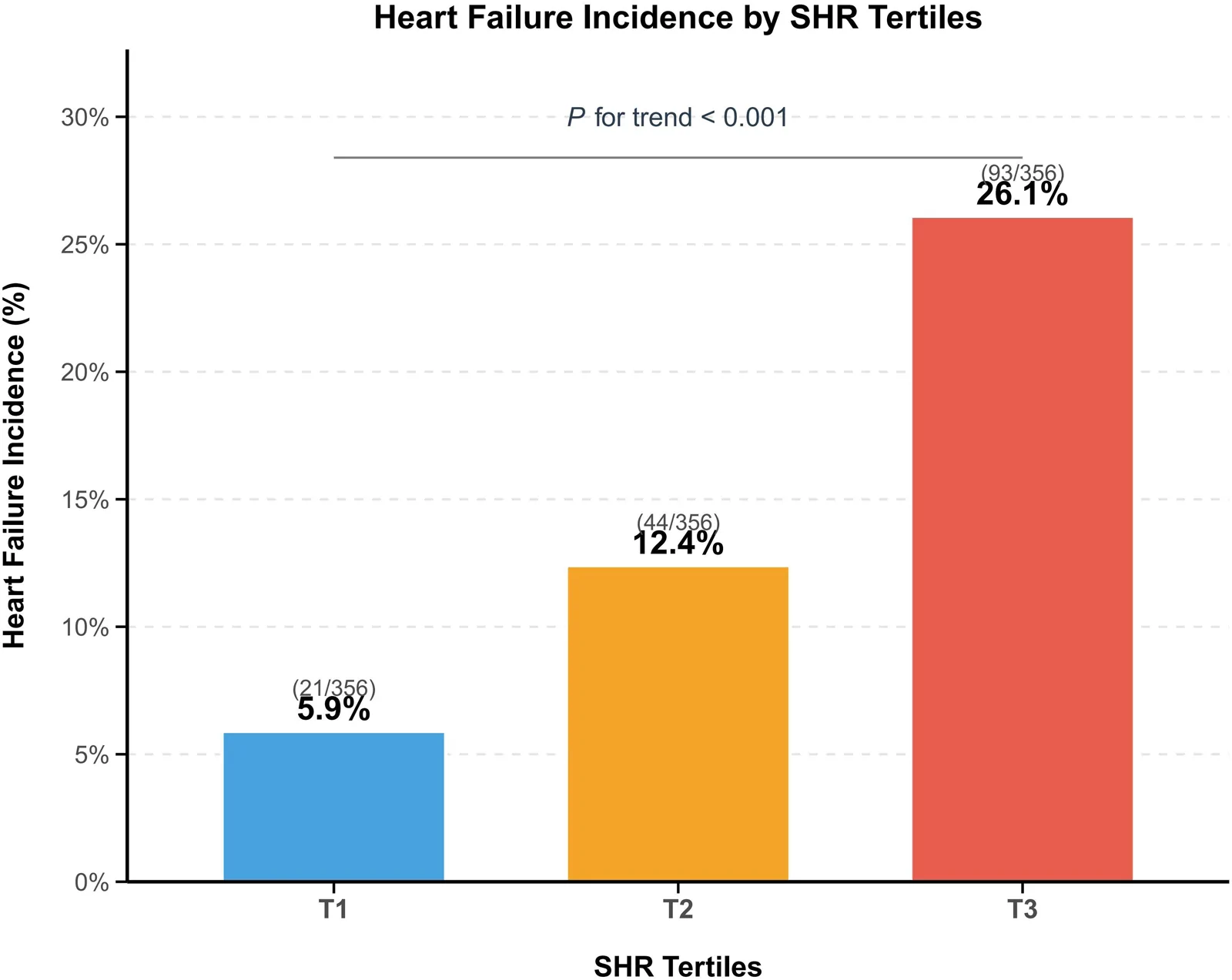
Trends In in-hospital heart failure incidence across three SHR tertile subgroups. *X*-axis labels represent the absolute SHR range for each tertile: T1 (0.30–0.98), T2 (0.99–1.28), T3 (1.29–5.00).

Patients were equally divided into three tertiles based on SHR distribution: T1 (SHR: 0.30–0.98, *n* = 356), T2 (SHR: 0.99–1.28, *n* = 356), and T3 (SHR: 1.29–5.00, *n* = 356). The incidence of in-hospital HF increased progressively across SHR tertiles: T1 = 5.9% (21/356), T2 = 12.4% (44/356), T3 = 26.1% (93/356) (*χ*^2^ for trend = 52.8, *P* for trend < 0.001; Figure 1).

### 3.3 Univariate and multivariate logistic regression analysis

**Figure 2:**
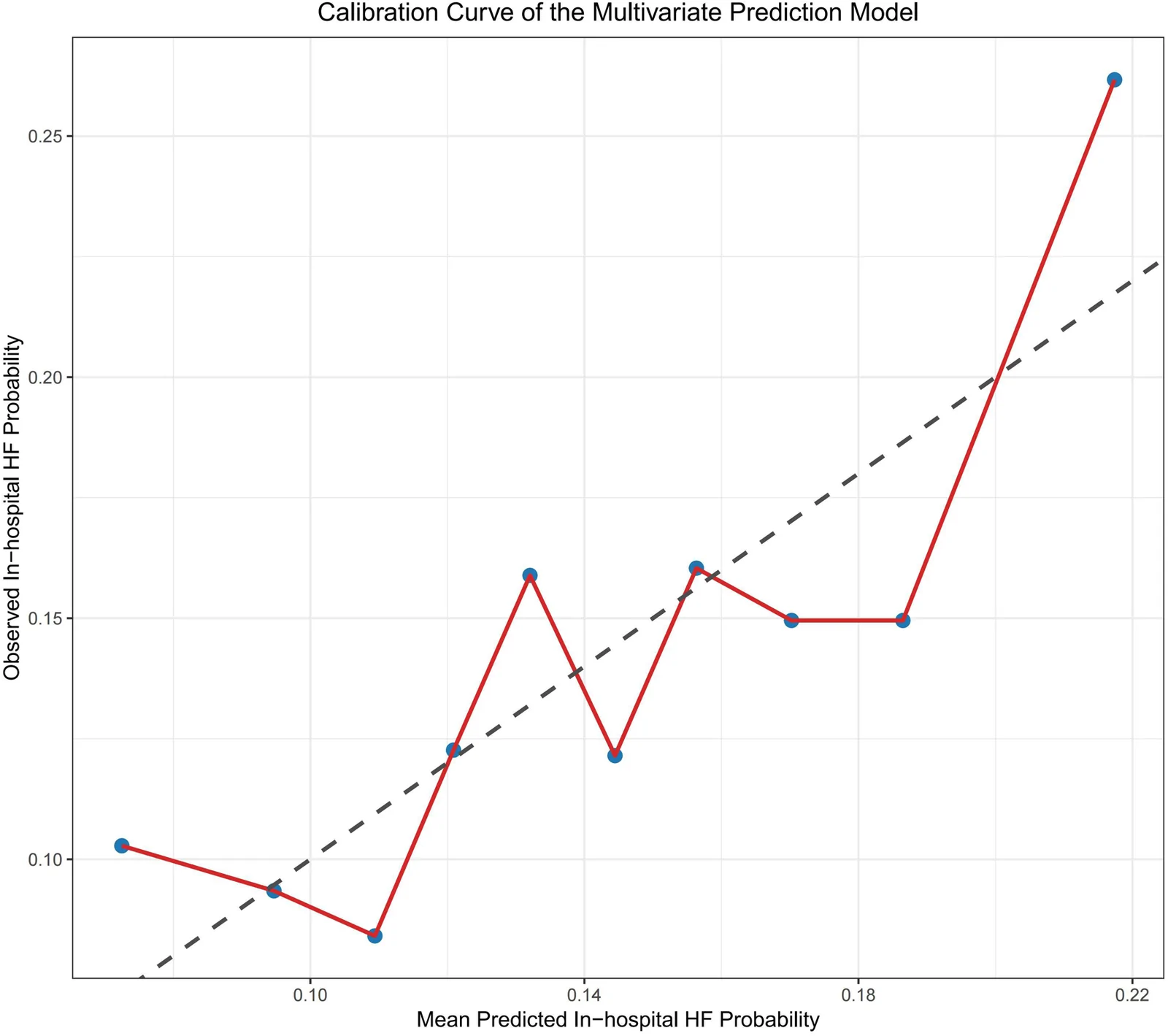
Calibration Curve of the multivariate logistic regression model for predicting in-hospital heart failure. The *x*-axis represents the mean predicted in-hospital HF probability stratified by ten equal risk deciles; the *y*-axis represents the actual observed in-hospital HF incidence in each risk decile. The dashed diagonal line indicates the ideal perfect calibration line where predicted probability equals observed probability. The scatter points and fitted curve show favorable agreement between predicted and observed event rates across all risk strata, confirming excellent calibration of the SHR-based predictive model.

In univariate analysis, each 1-unit increase in SHR was associated with a crude OR of 4.85 (95% CI 3.52–6.69; *P* < 0.001) for HF occurrence, representing the most significant effect size among all candidate predictors (Table 2). Variables with *P* < 0.05 in univariate analysis were entered into the multivariate logistic regression model. All included variables had VIF < 3.5, indicating no severe multicollinearity. The Hosmer-Lemeshow goodness-of-fit test yielded *χ*^2^ = 8.45, *P* = 0.391, indicating acceptable statistical calibration (Table 3). Further visual calibration analysis via risk decile stratification demonstrated excellent agreement between predicted and observed HF probabilities (Figure 2). The calibration curve showed no systematic overestimation or underestimation of in-hospital HF risk across low-, medium-, and high-risk patient subgroups, verifying the robust fitting performance of the multivariate predictive model. After multivariate adjustment, the independent predictive effect of SHR remained highly significant despite some attenuation from the crude OR: each 1-unit increase yielded an adjusted OR of 2.31 (95% CI 1.68–3.18; *P* < 0.001). In the tertile analysis, using T1 as the reference, T3 showed an adjusted OR of 2.86 (95% CI 1.62–5.05; *P* < 0.001), with *P* for trend < 0.001, indicating a significant “dose-response relationship” between SHR and HF risk. Within the same model, Killip class (adjusted OR=1.92 per grade) and LVEF (adjusted OR=0.92 per %) were also independent predictors, while hypertension and diabetes mellitus lost statistical significance after inclusion of SHR.

**Table 2:** Univariate logistic regression analysis of heart failure during hospitalization.

Variables	OR	95% CI	*P*-value
Age (per additional year)	1.04	1.03–1.06	< 0.001
Male	0.61	0.42–0.87	0.008
BMI (per additional 1 kg/m^2^)	1.05	0.99–1.11	0.085
Smoke	0.86	0.61–1.20	0.383
Hypertension	1.77	1.26–2.49	0.001
Diabetes	1.92	1.36–2.72	< 0.001
Dyslipidemia	1.25	0.87–1.78	0.223
Previous strok	1.87	1.08–3.22	0.025
Previous MI	1.83	0.98–3.40	0.057
Killip Grade (per additional 1 Grade)	2.85	2.38–3.41	< 0.001
Heart Rate (per additional 1 bpm)	1.04	1.03–1.05	< 0.001
SBP (per additional 1 mmHg)	0.98	0.97–0.99	< 0.001
LVEF (per additional 1%)	0.87	0.85–0.89	< 0.001
Multivessel disease	1.96	1.40–2.75	< 0.001
D-to-BTime (per additional 1 min)	1.003	1.001–1.005	0.003
Admission blood glucose (per additional 1 mmol/L)	1.18	1.14–1.22	< 0.001
SHR (per unit increase)	4.85	3.52–6.69	< 0.001
Peak cTnI (per additional 1 ng/mL)	1.02	1.01–1.02	< 0.001
NT-proBNP (per additional 100 pg/mL)	1.03	1.02–1.04	< 0.001
eGFR (per additional 1 mL/min/1.73 m^2^)	0.97	0.96–0.98	< 0.001
WBC (per additional 1 × 10⁹/L)	1.10	1.05–1.15	< 0.001
Hb (per additional 1 g/L)	0.97	0.96–0.98	< 0.001
LDL-C (per additional 1 mmol/L)	1.22	1.02–1.46	0.028
HDL-C (per additional 1 mmol/L)	0.38	0.19–0.76	0.006
Blood sodium (per additional 1 mmol/L)	0.92	0.87–0.96	< 0.001
UA (per additional 1 μmol/L)	1.004	1.002–1.006	< 0.001
D-dimer (per additional 1 mg/L)	1.18	1.10–1.27	< 0.001
hs-CRP (per additional 1 mg/L)	1.02	1.01–1.03	< 0.001

**Table 3:** Multivariate logistic regression analysis of heart failure during hospitalization.

Variables	Model 1:SHR continuous variables	*P*-value	Model 2:SHR tertiles	*P*-value
	Adjusted OR(95% CI)		Adjusted OR(95% CI)	
SHR (per unit)	2.31 (1.68–3.18)	< 0.001	—	—
SHR T1(References)	—	—	1.00(References)	—
SHR T2 vs. T1	—	—	1.62 (0.89–2.95)	0.112
SHR T3 vs. T1	—	—	2.86 (1.62–5.05)	< 0.001
Trend P	—	—	—	< 0.001
Age (per year)	1.02 (1.00–1.04)	0.038	1.02 (1.00–1.04)	0.042
Male	0.78 (0.48–1.26)	0.312	0.76 (0.47–1.24)	0.276
Hypertension	1.18 (0.78–1.80)	0.436	1.20 (0.79–1.83)	0.398
Diabetes	1.35 (0.86–2.12)	0.192	1.38 (0.88–2.17)	0.165
Killip grade (per grade)	1.92 (1.53–2.41)	< 0.001	1.95 (1.55–2.45)	< 0.001
Heart rate (per bpm)	1.01 (1.00–1.03)	0.068	1.01 (1.00–1.03)	0.072
SBP (per mmHg)	0.99 (0.98–1.00)	0.185	0.99 (0.98–1.00)	0.192
LVEF (per %)	0.92 (0.90–0.94)	< 0.001	0.92 (0.90–0.94)	< 0.001
Multivessel disease	1.28 (0.83–1.97)	0.262	1.30 (0.85–2.00)	0.228
D-to-B time (per min)	1.001 (0.999–1.003)	0.225	1.001 (0.999–1.003)	0.218
Peak cTnI (per ng/mL)	1.01 (1.00–1.01)	0.012	1.01 (1.00–1.01)	0.010
NT-proBNP (per 100 pg/mL)	1.01 (1.00–1.02)	0.005	1.01 (1.00–1.02)	0.006
eGFR (per mL/min/1.73 m^2^)	0.99 (0.98–1.00)	0.128	0.99 (0.98–1.00)	0.135
WBC (per × 10⁹/L)	1.03 (0.97–1.09)	0.318	1.03 (0.97–1.09)	0.325
Hb (per g/L)	0.99 (0.98–1.00)	0.218	0.99 (0.98–1.00)	0.225
HDL-C (per mmol/L)	0.62 (0.28–1.38)	0.245	0.60 (0.27–1.34)	0.218
Blood sodium (per mmol/L)	0.95 (0.90–1.01)	0.082	0.95 (0.90–1.01)	0.085
hs-CRP (per mg/L)	1.01 (1.00–1.02)	0.068	1.01(1.00–1.02)	0.072

### 3.4 Restrictive cubic spline analysis

**Figure 3:**
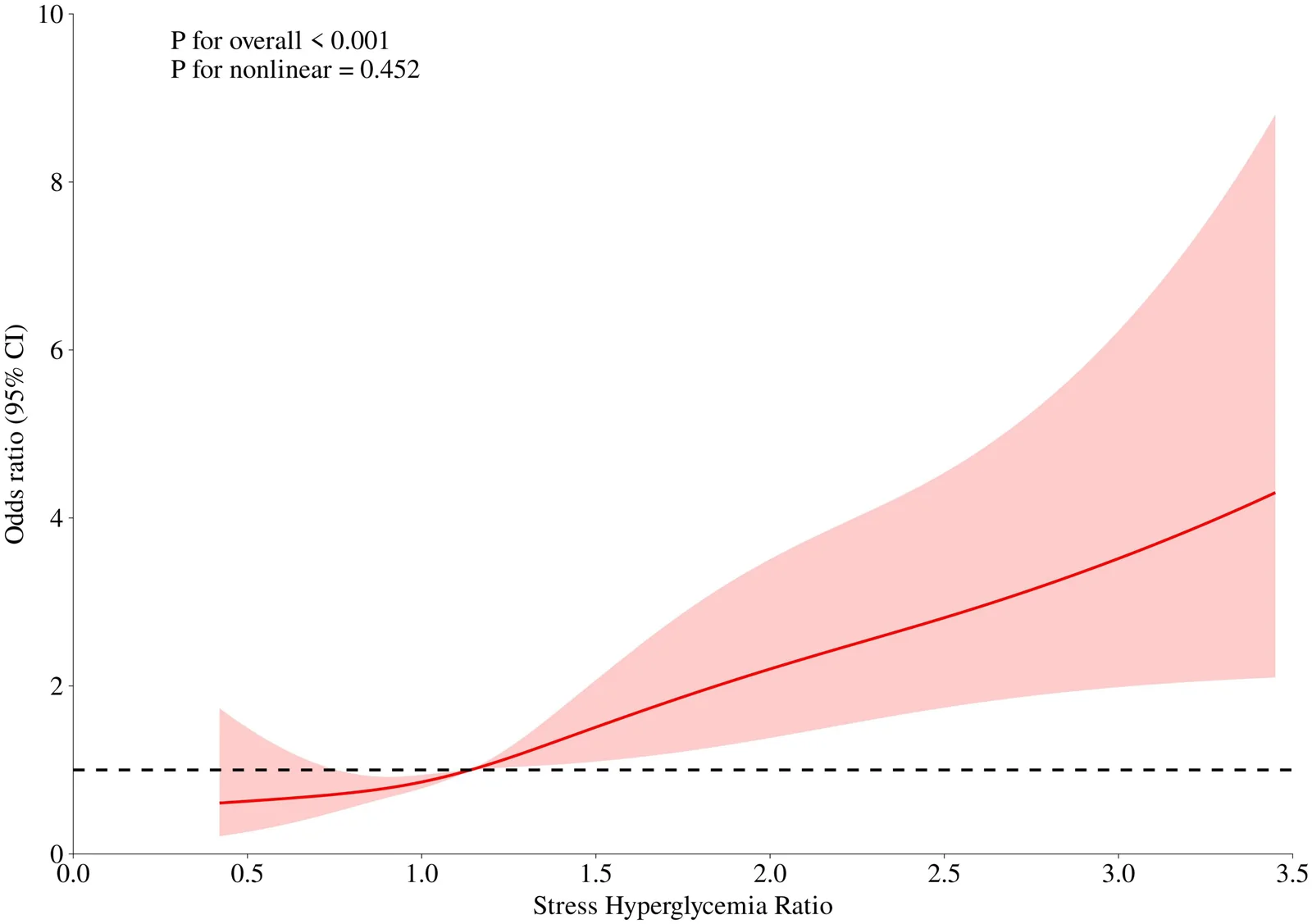
Dose-response Relationship between SHR and risk of in-hospital heart failure.

To explore the dose-response relationship between SHR and in-hospital heart failure risk, restricted cubic spline (RCS) regression analysis was performed. As shown in Figure 3, the RCS curve demonstrated a monotonically increasing trend: as SHR increased from 0.5 to 3.5, the adjusted OR progressively rose from below 1.0, indicating that higher SHR values were associated with greater risk of in-hospital heart failure. The overall association between SHR and heart failure was highly statistically significant (*P* overall < 0.001), while the test for nonlinearity was not significant (*P* nonlinear = 0.452), suggesting a linear dose-response relationship whereby each 1-unit increase in SHR was associated with a relatively constant proportional increase in heart failure risk, with no apparent threshold or plateau effects observed. Notably, when SHR exceeded approximately 1.2–1.3, the lower limit of the 95% confidence interval began to exceed the reference line (OR = 1.0), which closely aligned with the optimal cutoff value of 1.27 determined by the Youden index in ROC analysis. Consistent with visual observation, the confidence bands widened substantially at the extreme upper range of the SHR distribution (SHR > 2.5), which was attributed to data sparsity and limited sample size in the extreme high SHR subgroup.

### 3.5 Propensity score matching analysis

Patients were divided into high SHR (*n* = 534) and low SHR (*n* = 534) groups based on the median SHR value (1.14). Before matching, the two groups differed significantly in multiple baseline characteristics including age, diabetes mellitus, Killip class, and LVEF. After 1:1 nearest-neighbor PSM, 432 pairs (864 patients) were successfully matched. Post-matching, all covariates achieved absolute SMD < 0.10, indicating well-balanced covariates. After matching, the high SHR group had twice the incidence of in-hospital HF compared to the low SHR group (20.4% vs. 10.2%; OR=2.26, 95% CI 1.52–3.36; *P* < 0.001). Additionally, the high SHR group demonstrated significantly higher in-hospital mortality (3.7% vs. 1.2%; OR=3.28; *P* = 0.018) and longer length of stay [median 9 (7–13) vs. 7 (6–10) days; *P* < 0.001] compared to the low SHR group. The PSM results were highly consistent with the multivariate logistic regression findings, confirming the robustness of the association between SHR and HF (Table 4).

**Table 4:** Comparison of outcomes between the high SHR group and the Low SHR group after propensity score matching.

Outcome	High SHR Group (*n* = 432)	Low SHR Group(*n* = 432)	OR(95% CI)	*P*-Value
HF during hospitalization, *n*(%)	88 (20.4)	44 (10.2)	2.26 (1.52–3.36)	< 0.001
In-hospital death, *n*(%)	16 (3.7)	5 (1.2)	3.28 (1.19–9.03)	0.018
Days in hospital (days)^a^	9 (7–13)	7 (6–10)	—	< 0.001

a Non-normally distributed variables are expressed as median (IQR).

### 3.6 ROC curve analysis with optimal cutoff

**Figure 4:**
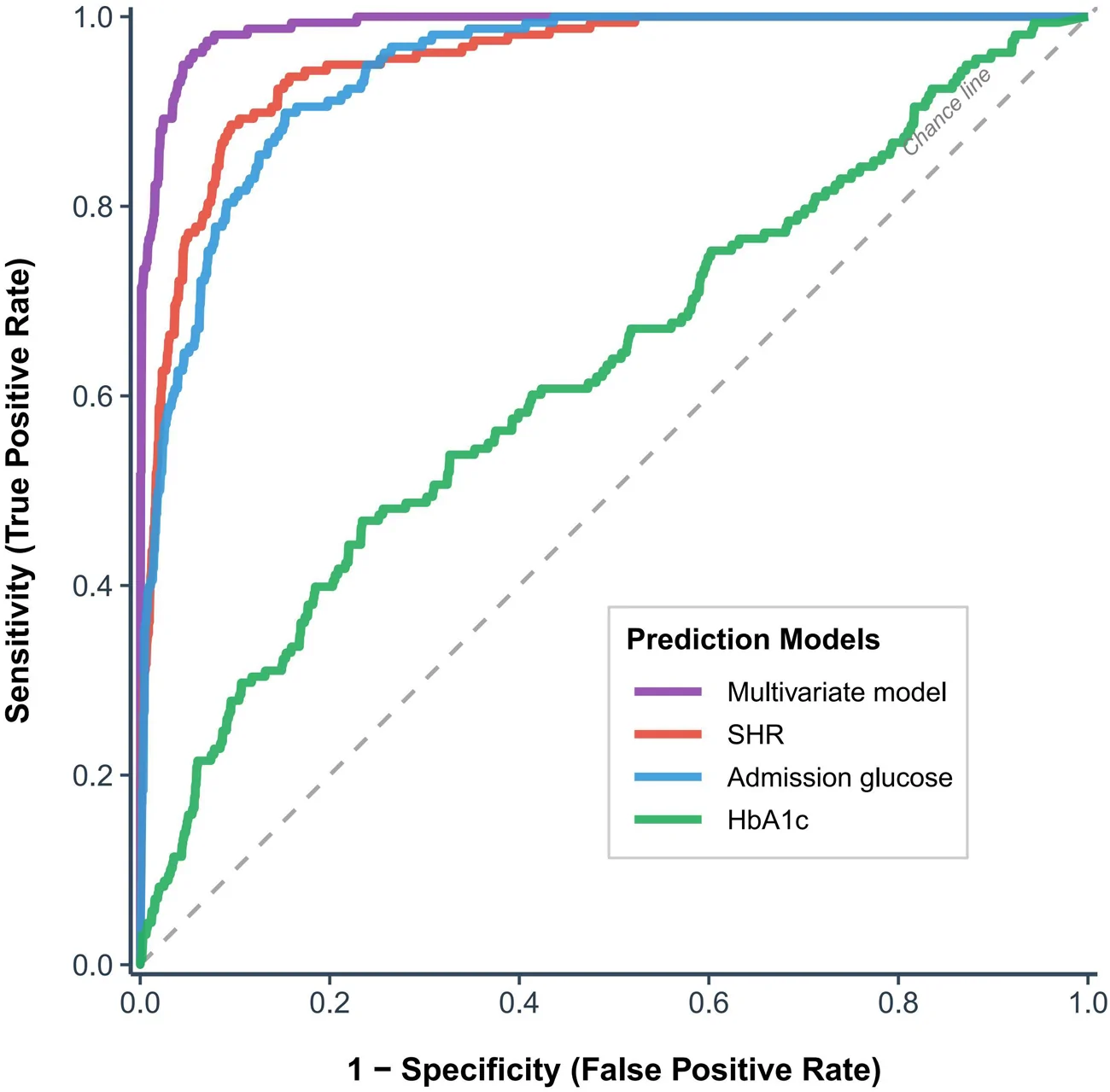
ROC Curve analysis.

The AUC of SHR for predicting in-hospital HF was 0.748 (95% CI 0.711–0.785), which was significantly superior to admission glucose (AUC = 0.728; DeLong *P* = 0.031) and HbA1c (AUC = 0.548; *P* < 0.001). The multivariate model integrating SHR with clinical covariates achieved a high AUC of 0.812 (95% CI 0.792–0.832). The optimal SHR cutoff determined by the Youden index was 1.27, corresponding to a sensitivity of 72.8%, specificity of 67.5%, PPV of 28.0%, and NPV of 93.4%. The high NPV (93.4%) indicates that SHR < 1.27 can effectively rule out in-hospital HF occurrence (Table 5). As shown in Figure 4, the ROC curve of SHR consistently positioned above and to the left of the admission glucose curve, while the HbA1c curve closely followed the diagonal reference line.

**Table 5:** ROC curve analysis for predicting heart failure during hospitalization.

Predictors	AUC (95% CI)	Cut-Off	Sensitivity (%)	Specificity (%)	PPV (%)	NPV (%)	vs. SHR DeLong P
SHR	0.748 (0.711–0.785)	1.27	72.8	67.5	28.0	93.4	—
Admission blood glucose	0.728 (0.690–0.766)	9.2 mmol/L	68.4	66.8	26.4	92.4	0.031
HbA1c	0.548 (0.503–0.593)	6.2%	55.1	52.8	16.9	87.2	< 0.001
Multivariate model	0.812 (0.792–0.832)	—	83.5	85.2	49.5	96.8	—

### 3.7 Incremental predictive value

After incorporating SHR into the baseline model containing conventional risk factors: AUC improved from 0.898 to 0.912 (*Δ*AUC =  + 0.014; *P* = 0.008); likelihood ratio test *χ*^2^ = 18.6, *P* < 0.001; continuous NRI = 0.318 (*P* < 0.001), with event NRI = + 0.224 indicating that 22.4% of HF patients were correctly reclassified to higher risk categories, and non-event NRI = + 0.094 indicating that 9.4% of non-HF patients were correctly reclassified to lower risk categories; IDI = 0.035 (*P* < 0.001). The SHR-containing model demonstrated lower AIC (645.2) and BIC (724.8) compared to both the baseline model (662.8/742.4) and the admission glucose model (658.7/738.3), supporting superior model fit for SHR (Table 6).

**Table 6:** Incremental predictive value of SHR beyond the basic clinical model.

Assessment indicators	Value	95%CI/*P*-value
Base model AUC	0.898	0.876–0.920
Base model + SHR AUC	0.912	0.892–0.932
*Δ*AUC	+0.014	DeLong *P* = 0.008
Likelihood ratio test *χ*^2^	18.6	*P* < 0.001
Categoryless NRI	0.318	*P* < 0.001
NRI(event)	+0.224	—
NRI(non-event)	+0.094	—
IDI	0.035	*P* < 0.001
Base model AIC	662.8	—
Base model + SHR AIC	645.2	—
Base model + admission blood glucose AIC	658.7	—
Base model BIC	742.4	—
Base model + SHR BIC	724.8	—
Base model + admission blood glucose BIC	738.3	—

The basic clinical model was adjusted for all conventional prognostic risk factors, including age, sex, body mass index, smoking history, alcohol consumption, hypertension, diabetes mellitus, dyslipidemia, previous ischemic stroke, previous myocardial infarction, previous PCI history, heart rate, systolic blood pressure, diastolic blood pressure, peak cardiac troponin I, NT-proBNP, eGFR, white blood cell count, high-sensitivity C-reactive protein, D-dimer, left ventricular ejection fraction, culprit vessel type, multivessel disease, door-to-balloon time, and post-procedural TIMI flow grade. This baseline model did not include SHR or admission glucose.

### 3.8 Subgroup analysis and sensitivity analysis

**Figure 5:**
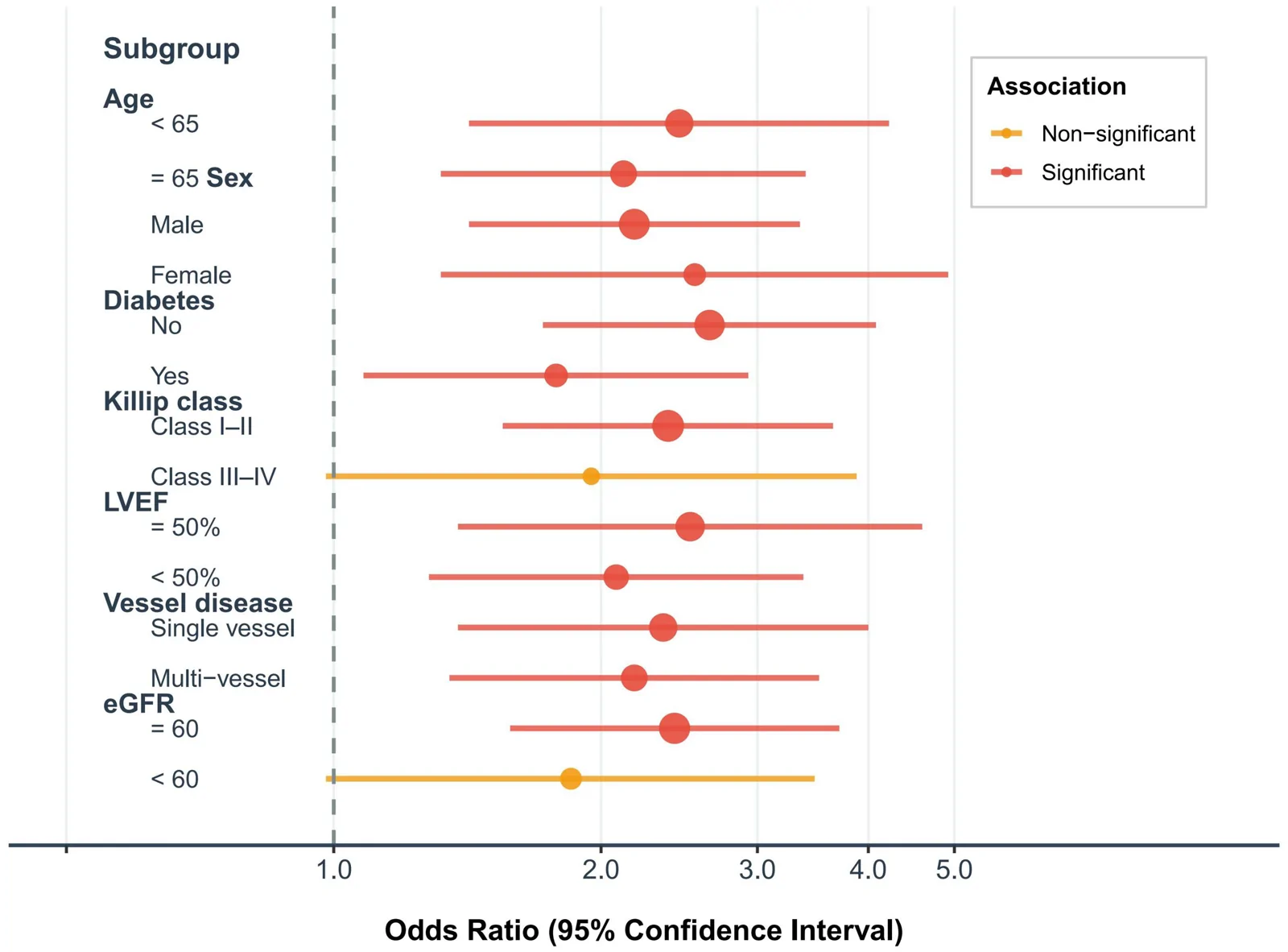
Forest Plot for subgroup analyses.

As shown in the forest plot (Figure 5), the association between elevated SHR and increased risk of in-hospital HF was consistent in direction across all predefined subgroups (all ORs > 1). All interaction *P* values were > 0.05, indicating that the predictive effect of SHR was not significantly modified by age, sex, LVEF level, extent of coronary disease, or renal function status. Notably, there was a borderline interaction trend for diabetes status (interaction *P* = 0.068): the effect size of SHR was numerically greater in non-diabetic patients (OR=2.65) than in diabetic patients (OR=1.78), though the 95% CIs of both groups overlapped (Table 7). Across various sensitivity analyses, the independent predictive effect of SHR remained robust: adjusted ORs fluctuated between 1.85–2.58, with consistent direction and significance (Table 8).

**Table 7:** Subgroup analysis: association between high SHR (≥median) and heart failure during hospitalization.

Subgroups	*n*	Number of events	OR(95% CI)	*P*-value	Interaction *p*-value
Age					0.385
<65	592	62	2.45 (1.42–4.22)	0.001	
≥65	476	96	2.12 (1.32–3.40)	0.002	
Gender					0.528
Male	820	108	2.18 (1.42–3.35)	< 0.001	
Female	248	50	2.55 (1.32–4.92)	0.005	
Diabetes					0.068
No	780	98	2.65 (1.72–4.08)	< 0.001	
Yes	288	60	1.78 (1.08–2.93)	0.024	
Killip Grade					0.312
Ⅰ–Ⅱ	924	103	2.38 (1.55–3.65)	< 0.001	
Ⅲ–Ⅳ	144	55	1.95 (0.98–3.88)	0.058	
LVEF					0.425
≥50%	685	52	2.52 (1.38–4.60)	0.003	
<50%	383	106	2.08 (1.28–3.38)	0.003	
Vascular Lesions					0.562
Single branch	598	66	2.35 (1.38–4.00)	0.002	
Multiple branches	470	92	2.18 (1.35–3.52)	0.002	
eGFR					0.218
≥60	852	108	2.42 (1.58–3.71)	< 0.001	
<60	216	50	1.85 (0.98–3.48)	0.056	

**Table 8:** Sensitivity analysis.

Analysis	n	Adjusted OR (95% CI)	*P*-value
Main analysis (full cohort)	1 068	2.31 (1.68–3.18)	< 0.001
Non-diabetic subgroup	780	2.58 (1.72–3.87)	< 0.001
Diabetic subgroup	288	1.85 (1.12–3.05)	0.016
Exclude patients with Killip IV	1 023	2.25 (1.62–3.12)	< 0.001
Exclude eGFR <30	1 042	2.28 (1.65–3.15)	< 0.001
PSM matching cohort	864	2.26 (1.52–3.36)	< 0.001

## 4 Discussion

In this single-center cross-sectional study including 1,068 STEMI patients undergoing emergent PCI, we systematically evaluated the predictive value of the stress hyperglycemia ratio (SHR) for in-hospital heart failure. The main findings were as follows: SHR was a strong independent predictor of in-hospital HF. After multivariate adjustment for covariates, each 1-unit increase in SHR was associated with a 2.31-fold increase in HF risk (adjusted OR = 2.31, 95% CI 1.68–3.18). The highest SHR tertile (T3) demonstrated nearly 3-fold higher HF risk compared to the lowest tertile (T1) (adjusted OR = 2.86), with a significant dose-response trend (*P* for trend < 0.001). A linear dose-response relationship existed between SHR and HF risk. RCS analysis revealed that each 1-unit increase in SHR was associated with a relatively constant proportional increase in heart failure risk, with no apparent threshold or plateau effects observed. The SHR-HF association demonstrated robustness through propensity score matching validation. After matching, the high SHR group maintained more than twice the HF incidence of the low SHR group (20.4% vs. 10.2%). SHR showed superior discriminatory performance compared to conventional glycemic indices. The AUC for SHR (0.748) was significantly higher than that of admission glucose (0.728; *P* = 0.031) and HbA1c (0.548; *P* < 0.001). SHR provided significant incremental predictive value. When added to the baseline model, NRI = 0.318 and IDI = 0.035 (both *P* < 0.001), with superior AIC and BIC compared to the admission glucose model. The predictive effect of SHR remained consistent across all predefined subgroups, unmodified by age, sex, diabetes status, LVEF level, extent of coronary disease, or renal function status.

Our findings are consistent with and extend recent literature on the prognostic value of SHR in acute cardiovascular events. Yang et al. (14) reported that elevated SHR was an independent predictor of MACE during a median follow-up of 2.1 years in 1,262 AMI patients undergoing PCI (highest vs. lowest quartile HR = 1.89). Xu et al. (15) found that SHR could predict no-reflow phenomenon and in-hospital mortality in STEMI patients. Gao et al. (16), based on MIMIC-IV database analysis, demonstrated that SHR was superior to admission glucose in predicting 30-day and 1-year mortality after AMI.

However, most previous studies focused on composite MACE endpoints or long-term mortality, with few specifically investigating in-hospital HF as an independent primary endpoint. This study is the first to systematically examine in-hospital HF as the primary endpoint, integrating multivariate regression, PSM, RCS, and incremental predictive value analyses to provide the most comprehensive evidence to date regarding the SHR-in-hospital HF association. The observed in-hospital HF incidence (14.8%) in our study was consistent with contemporary PCI-era registry studies. Bahit et al. reported a 13.8% in-hospital HF incidence in the STEMI subgroup of the PLATO trial, while Desta et al. reported 16.2% in the SWEDEHEART registry study. Our data fall within this range, suggesting representativeness of our study population.

The association between SHR and post-STEMI HF has a solid pathophysiological foundation involving multiple interconnected mechanisms: Acute hyperglycemia induces cardiomyocyte injury through multiple pathways: ① excessive superoxide anion (O₂⁻) production by the mitochondrial electron transport chain, initiating oxidative stress cascades (24); ② activation of the polyol and hexosamine pathways, leading to increased protein O-GlcNAc modifications that interfere with calcium-regulating protein function (25); ③ promotion of advanced glycation end product (AGE) formation and their receptor (RAGE) signaling, triggering pro-fibrotic and pro-apoptotic gene expression (26). In the context of ischemia-reperfusion (I/R), hyperglycemia exacerbates reperfusion injury by attenuating ischemic preconditioning and lowering the threshold for mitochondrial permeability transition pore (mPTP) opening (27). Stress hyperglycemia impairs microcirculatory perfusion by reducing nitric oxide (NO) bioavailability, increasing endothelin-1 (ET-1) expression, and upregulating vascular cell adhesion molecule-1 (VCAM-1) (28). Even after successful epicardial vessel recanalization, microvascular obstruction (MVO) can persist, leading to expansion of the effective infarct area and ventricular remodeling.

Elevated SHR essentially reflects the severity of acute stress response: excessive sympatho-adrenal system activation results in dramatic increases in circulating catecholamine concentrations, which directly increase myocardial oxygen consumption and arrhythmia risk while causing myocardial stunning through *β*1-adrenergic receptor-mediated calcium overload (29). HPA axis activation leads to cortisol surges, further exacerbating insulin resistance and hyperglycemia. Furthermore, acute hyperglycemia can directly activate inflammatory signaling pathways including NF-*κ*B (30), Toll-like receptor-4 (TLR-4), and the NLRP3 inflammasome (31), driving the cascading release of interleukin-1β (IL-1β), interleukin-6 (IL-6), and tumor necrosis factor-α (TNF-α). These pro-inflammatory mediators not only aggravate acute-phase myocardial injury but also promote chronic fibrosis and adverse remodeling, forming the pathological bridge from acute injury to heart failure.

Hyperglycemia enhances platelet GPIIb/IIIa receptor expression and platelet aggregation activity (32), upregulates tissue factor (TF) expression (33), and inhibits fibrinolysis by elevating plasminogen activator inhibitor-1 (PAI-1) levels (34). These prothrombotic effects may promote microvascular thrombosis, in-stent thrombosis, and infarct expansion. The normal heart primarily relies on fatty acid *β*-oxidation for ATP generation, but shifts toward more “oxygen-efficient” aerobic and anaerobic glucose metabolism under ischemic conditions (35). Acute insulin resistance (a core feature of elevated SHR) severely impairs glucose uptake capacity in ischemic myocardium by inhibiting glucose transporter 4 (GLUT-4) translocation to the cell membrane, forcing the myocardium to be “deprived” of this critical substrate precisely when glucose is most needed this “metabolic inflexibility” represents an important mechanism leading to further deterioration of contractile function and pump failure (36).

Notably, the widened confidence intervals in the high extreme SHR range in RCS analysis deserve explicit interpretation. The substantial broadening of confidence bands for SHR values greater than 2.5 is caused by data sparsity in the upper tail of the SHR distribution, where only a small number of patients presented with extremely elevated stress hyperglycemia. This sparse data distribution increases statistical sampling uncertainty and reduces the model's quantitative predictive reliability for extreme high-risk outliers. Nevertheless, the tightly narrowed and stable confidence intervals across the main SHR distribution range (0.5–2.5) firmly support the robust linear dose-response relationship between SHR and in-hospital HF risk in the vast majority of STEMI patients. This limitation only affects extreme outliers rather than the overall core conclusion of the study.

The AUC of SHR (0.748) was significantly superior to that of admission glucose (0.728; *P* = 0.031) and HbA1c (0.548; *P* < 0.001). Notably, as a derived index calculated from admission glucose and HbA1c, SHR has inherent interpretative limitations in ROC analysis, and its predictive efficacy cannot be completely decoupled from the two original glycemic indicators. The superior discriminative ability of SHR does not originate from novel independent biomarker information, but from the individualized standardization of admission glucose based on patients’ chronic glycemic status, which effectively removes the confounding effect of baseline glucose metabolism and improves the accuracy of acute stress hyperglycemia evaluation. Consistent with our ROC findings, subsequent incremental predictive value analyses further confirmed that SHR provided significant NRI and IDI for HF risk stratification compared with raw admission glucose, with better model fitness (lower AIC and BIC). These results collectively demonstrate that SHR is a clinically optimized glycemic index rather than a simply mathematically derived variable, with unique practical value for identifying post-PCI HF high-risk patients that cannot be replaced by absolute admission glucose levels.

This study has the following limitations that warrant consideration: First, this single-center retrospective study had inherent data limitations. Detailed electrocardiography-based STEMI anatomical location was not uniformly documented in electronic medical records during the enrollment period; therefore, we adopted culprit vessel type and multivessel disease as objective alternative indicators to reflect infarct-related ischemic characteristics, which were fully adjusted in multivariate models. In addition, dynamic myocardial enzyme measurements were not incorporated due to inconsistent admission and blood-sampling time windows among emergency STEMI patients with heterogeneous disease onset times. Instead, uniformly recorded peak cTnI was used to quantitatively reflect the maximum severity of myocardial necrosis, which is the most reliable biomarker for evaluating myocardial injury and predicting post-procedural heart failure. Future prospective studies with standardized unified sampling time points and complete infarct location recording are needed to further validate our findings. Second, admission glucose represented a single measurement (at the emergency department time point) and may not fully represent glycemic status throughout hospitalization. Continuous glucose monitoring (CGM) or multi-timepoint glucose measurements might provide more comprehensive glucose exposure information. Third, HbA1c as the basis for eAG derivation has limitations: in patients with concurrent anemia, hemoglobinopathies, recent blood transfusions, or severely impaired hepatic or renal function, HbA1c may not accurately reflect chronic glycemic levels [40]. Although this study excluded extreme HbA1c values (< 4.0% and > 14.0%) and patients with severe hepatic dysfunction, these confounders could not be completely eliminated. Fourth, the definition of in-hospital HF, while incorporating multiple dimensions (Killip class, clinical diagnosis, imaging, and hemodynamics), inevitably involves information bias due to its retrospective determination. Prospective studies should employ standardized HF diagnostic protocols and core laboratory adjudication. Fifth, the lack of external validation cohorts represents a limitation. The optimal cutoff value (SHR=1.27) and predictive model derived from this study require external validation in independent multicenter cohorts before widespread application. Sixth, restricted cubic spline analysis revealed markedly widened 95% confidence bands at the extreme upper tail of the SHR distribution (SHR > 2.5), which is attributed to obvious data sparsity and limited sample size among patients with extremely high SHR values. Such sparse data distribution increases statistical sampling uncertainty and reduces the quantitative predictive accuracy of the model for extreme high-risk outliers. Nevertheless, the confidence intervals remained narrow and stable across the major SHR distribution range (0.5–2.5), strongly supporting the robust linear dose-response relationship between SHR and in-hospital HF risk in the vast majority of STEMI patients; this limitation only affects extreme outliers and does not compromise the core conclusion of the present study. In addition, since SHR is a secondary derived index based on admission glucose and HbA1c, its ROC predictive performance has certain interpretative limitations and cannot provide completely independent biomarker information beyond the original glycemic indicators. The superior AUC of SHR relative to admission glucose only reflects the prognostic improvement effect of chronic glycemic calibration, rather than the discovery of a new predictive biomarker. Future studies are warranted to explore novel independent stress biomarkers to further refine risk stratification for post-STEMI HF.

In conclusion, the present study demonstrates that SHR is a robust, independent predictive biomarker for in-hospital heart failure in STEMI patients undergoing emergency PCI. By eliminating the confounding interference of individual chronic glycemic status, SHR exhibits superior predictive accuracy and incremental prognostic value compared with conventional admission glucose. The linearly increased HF risk alongside elevated SHR, as well as the consistent predictive efficacy across diverse clinical subgroups, further validates its reliable clinical application value. With a favorable discriminatory ability and clear risk stratification cutoff, SHR can serve as a convenient and effective tool for early in-hospital risk assessment. Routine calculation of SHR at admission may help clinicians identify high-risk STEMI patients who are vulnerable to post-PCI heart failure, formulate individualized intensive monitoring and intervention strategies, and ultimately improve the in-hospital clinical prognosis of STEMI patients.

## Data Availability

The original contributions presented in the study are included in the article/Supplementary Material, further inquiries can be directed to the corresponding author.
